# Supplementary material for: First-Line Treatment for Primary Breast Diffuse Large B-Cell Lymphoma Using Immunochemotherapy and Central Nervous System Prophylaxis: A Multicenter Phase 2 Trial
Source: Cancers (Basel). 2020 Aug 6;12(8):2192. doi: 10.3390/cancers12082192 (PMC7463683; doi:10.3390/cancers12082192)
Supplement: Supplementary file 1 [file cancers-12-02192-s001.pdf]

Supplementary Materials: First-Line Treatment for Primary Breast Diffuse Large B-Cell Lymphoma Using Immunochemotherapy and Central Nervous System Prophylaxis: A Multicenter Phase 2 Trial

Ho-Young Yhim, Dok Hyun Yoon, Seok Jin Kim, Deok-Hwan Yang, Hyeon-Seok Eom, Kyoung Ha Kim, Yong Park, Jin Seok Kim, Hyo Jung Kim, Cheolwon Suh, Won Seog Kim and Jae-Yong Kwak

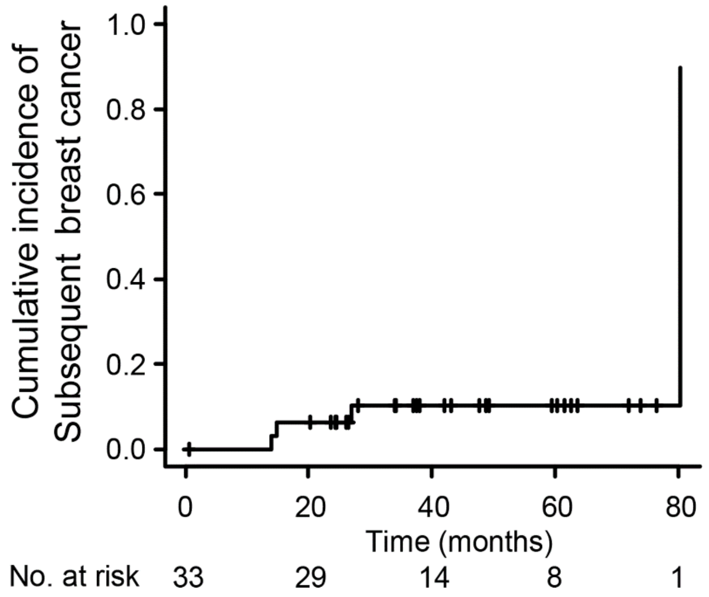

Figure S1. Cumulative incidence of subsequent breast cancer. The 3-year cumulative incidence of subsequent breast cancer, taking into account the competing risk of death, was 10.2% (95% CI, 2.5-24.6). Tick marks indicate censored observations.

Table S1. 2-year cumulative incidence according to baseline clinical variables.

Variables	Patients (n, %)	2-year cumulative incidence of CNS relapse	
		% (95% CI)	p
Age, years			
≤60	24 (72.7)	12.5 (0.9-24.8)	0.954
>60	9 (27.3)	12.5 (1.6-32.6)	
Ann Arbor stage			
IE	17 (51.5)	11.7 (0-25.8)	0.896
IIE - IV	16 (48.5)	13.3 (0-28.9)	
B symptoms			
Absence	31 (93.9)	10.0 (0.6-20.1)	0,071
Presence	2 (6.1)	50.0 (0-87.5)	
Serum LDH level			
Normal	24 (72.7)	12.5 (0.9-24.8)	0.954
Increased	9 (27.3)	12.5 (1.6-32.6)	
Bulky disease			
No	32 (97.0)	12.9 (0.2-23.9)	0.706
Yes	1 (3.0)	NA	
CNS-IPI			
Low	28 (84.8)	10.7 (0-21.5)	0.379
Intermediate	5 (15.2)	25.0 (0-57.4)	

Abbreviations: CNS, central nervous system; LDH, lactate dehydrogenase; NA, not available; CNS-IPI, central nervous system-international prognostic index.

© 2020 by the authors. Licensee MDPI, Basel, Switzerland. This article is an open access article distributed under the terms and conditions of the Creative Commons Attribution (CC BY) license (http://creativecommons.org/licenses/by/4.0/).
